# The Role of Glycerol-Containing Drugs in Cerebral Microdialysis: A Retrospective Study on the Effects of Intravenously Administered Glycerol

**DOI:** 10.1007/s12028-018-0643-4

**Published:** 2018-11-14

**Authors:** Maximilian Peter Forssten, Eric Peter Thelin, David W. Nelson, Bo-Michael Bellander

**Affiliations:** 1 Department of Clinical Neuroscience, Section for Neurosurgery, Karolinska Institutet, Karolinska University Hospital, Stockholm, Sweden; 2 Division of Neurosurgery, Department of Clinical Neurosciences, University of Cambridge, Cambridge, UK; 3 Department of Physiology and Pharmacology, Karolinska Institutet, Stockholm, Sweden

**Keywords:** Microdialysis, Glycerol, Neurophysiological monitoring, Intensive care

## Abstract

**Background:**

Cerebral microdialysis (CMD) is a valuable tool for monitoring compounds in the cerebral extracellular fluid (ECF). Glycerol is one such compound which is regarded as a marker of cell membrane decomposition. Notably, in some acutely brain-injured patients, CMD-glycerol levels rise without any other apparent indication of cerebral deterioration. The aim of this study was to investigate whether this could be due to an association between CMD-glycerol levels and the administration of glycerol-containing drugs.

**Methods:**

Microdialysis data were retrospectively retrieved from the hospital’s intensive care unit patient data management system (PDMS). All patients who were monitored with CMD for ≥ 96 h were included. Administered drug doses were retrieved from the PDMS and converted to exact doses of glycerol. Cross-correlation analyses were performed between the free, metabolized as well as total administered dose of glycerol and the detrended and differenced CMD-glycerol concentration. These analyses were repeated for two sets of subgroups based upon the individual catheter’s graphical trend and its location in relation to the lesion.

**Results:**

There was no significant correlation between the differenced CMD-glycerol levels and drug-administered glycerol. Furthermore, there was no significant correlation between CMD-glycerol and catheter location or graphical trend. However, if the CMD-glycerol levels were detrended, significant but clinically non-relevant correlations were identified (maximum correlation coefficient of 0.1 (0.04–0.15, 95% CI) at a lag of 7 h using the total administered dose of glycerol).

**Conclusions:**

Glycerol-containing drugs routinely administered intravenously in the clinical setting appear to have a minimal and clinically insignificant effect on levels of glycerol in the cerebral ECF.

## Introduction

Microdialysis is a technique used for monitoring the concentration of compounds within the extracellular fluid (ECF) [1]. Today, cerebral microdialysis (CMD) is used in several neurocritical care units (NCCU) [2] to monitor patients with traumatic brain injuries (TBI) [2, 3] or subarachnoid hemorrhages [2]. Clinical CMD assesses the cerebral metabolism by measuring the concentrations of glucose, lactate, pyruvate, glutamate, glycerol and calculating the lactate/pyruvate ratio (LPR) [1–4].

In the traumatized brain, imbalance in the supply and demand of energetic substrates and oxygen may cause cerebral ischemia, leading to an influx of calcium into cells and degradation of phospholipids [1, 4, 5], which releases glycerol into the ECF [2]. Therefore, CMD-glycerol is considered an indicator for ongoing cell membrane degradation [2] and has been used in conjunction with the LPR in order to predict imminent patient deterioration [5], albeit the association with outcome is weak [3].

The interpretation of glycerol changes in the ECF is complex and may also be confounded by extracranial sources [1, 4]. Activation of the sympathetic nervous system results in an increased lipolysis of triglycerides in adipose tissue, which raises the systemic and possibly the cerebral levels of glycerol independently of cell membrane decomposition. A catheter inserted in the subcutaneous periumbilical region may be used to adjust for this [1]. Despite this, general patterns have been observed that cannot with certainty be coupled to ongoing cell death or systemic release. In some patients, after a few days of CMD monitoring in the NCCU, the CMD-glycerol appears to increase without any other apparent signs of deterioration. The explanation for this increasing trend of CMD-glycerol is poorly understood.

Although CMD-glycerol appears to primarily originate from local release in the brain [6], it is also known to be affected by the administration of glycerol-containing substances [4]. A study on rabbits displayed that glycerol could pass through, albeit not freely, an intact blood–brain barrier (BBB) [7]. Furthermore, administration of glycerol-containing enemas has been shown to significantly increase CMD-glycerol levels [8, 9]. However, no studies have been performed concerning the effect of intravenously administered glycerol on the cerebral glycerol concentration. It is therefore worth noting that drugs containing a glycerol vehicle such as propofol and parenteral nutrition are commonplace in the NCCU; consequently, we hypothesized that a correlation may exist between intravenously administered glycerol and the CMD-glycerol levels. Our aim was therefore to determine whether such a relationship exists in a retrospective cohort of NCCU patients monitored with CMD.

## Methods

### Study Design

All CMD data were retrospectively retrieved from the hospital’s intensive care unit patient data management system (PDMS; Clinisoft^®^, Centricity™, GE Healthcare, Chicago, IL, USA), restricted to all patients who had been monitored with CMD in the NCCU at the Karolinska University Hospital, Stockholm, Sweden, for a total of at least 96 h between January 1, 2006, and April 13, 2017. The PDMS registered all drugs patients received; by cross-referencing these drugs with their compositions provided by the manufacturers, we identified and retrieved the doses for all intravenously administered glycerol-containing drugs (propofol, insulin, parenteral nutrition, and Glycophos^®^).

### Collection of CMD Data

CMD catheters were predominantly inserted at admission to the NCCU and aimed for pericontusional areas in TBI patients while usually being placed in white matter in the right frontal lobe of patients with non-focal injuries. CMA70, CMA71, and CMA64 catheters (µDialysis AB, Stockholm, Sweden) were used with a membrane length of 10 mm.

The CMA70 and CMA71 were both inserted in the cerebral parenchyma with a flow rate of 0.3 μL/min, but differ in pore size, 20 kDa and 100 kDa, respectively. These catheters have been shown to yield equivalent results for glucose, lactate, pyruvate, glutamate, and LPR [10]. The CMA64 (20 kDa) was used for “global microdialysis” inserted in an external ventricular drain to assess glucose, lactate, pyruvate, and LPR in CSF extracted at a rate of 2 mL/h [6].

CMD-glycerol was analyzed hourly, using the CMA 600 Microdialysis Analyzer (µDialysis AB, Stockholm, Sweden) or ISCUSflex Microdialysis Analyzer (µDialysis AB, Stockholm, Sweden). The CMA 600 Microdialysis Analyzer was used until July 26, 2016. The ISCUSflex Microdialysis Analyzer was used concurrently with the CMA 600 starting on April 2, 2012, and exclusively after July 26, 2016. Using the same enzymatic reagents supplied by the manufacturer, these devices measure the concentrations of glucose, lactate, pyruvate, and glycerol, which is then automatically registered in the PDMS.

### Type of Data

#### Time Series of CMD-Glycerol

The CMD-glycerol data were used to create a time series for each catheter containing the CMD-glycerol level every hour. In some cases, patients had two microdialysis catheters simultaneously (*n* = 28; 9 in the parenchyma, 19 in the CSF). These catheters were considered independent in the respect that each catheter demonstrated a unique trend unrelated to the other catheter in the same patient; however, while each catheter was analyzed separately, the analyses used the same administered doses of glycerol since those were unique to each patient and not each catheter.

#### Time Series of Administered Dose of Glycerol

The administered dose of glycerol (ADG) was determined by multiplying the concentration of glycerol in each specific drug with the volume of that drug administered. All the ADG given during the same hour was then summated to establish the overall hourly ADG.

Three different ADGs were calculated: (1) the dose of free glycerol, the amount of glycerol in the drug that was not a constituent of any other compounds, (2) the dose of metabolized glycerol, the estimated amount of glycerol released through metabolism of glycerol-containing compounds in the drugs (triglycerides, egg lecithin, and sodium glycerophosphate), and (3) the total dose of glycerol, the sum of the dose of free and metabolized glycerol. These are referred to as free, metabolized, and total ADG, respectively. The concentration of free glycerol and the information for calculating the dose of metabolized glycerol were supplied by the drug manufacturers.

### Statistical Analysis

For each catheter, a cross-correlation analysis was performed between each of the ADG time series and the CMD-glycerol time series using the R statistical programming language [11]. In a cross-correlation analysis, the Pearson correlation coefficient is calculated between two time series at consecutively larger time delays, or lag values, by shifting one time series relative to the other. In this case, we selected a maximum time delay of 20 h between the administration of glycerol and measurement of CMD-glycerol; we considered that this would provide a more than adequate time frame for the administered glycerol to reach the cerebral ECF. Once all the analyses for each individual catheter were completed, the mean correlation coefficient at each lag value along with the 95% confidence interval could be calculated.

The cross-correlation analysis has two advantages. Primarily, it allows us to establish if a correlation exists. Additionally, the amount of time it takes for the administered glycerol to reach the cerebral ECF can be determined based upon the time delay that has the largest correlation coefficient.

#### Detrended Analysis

**Fig. 1:**
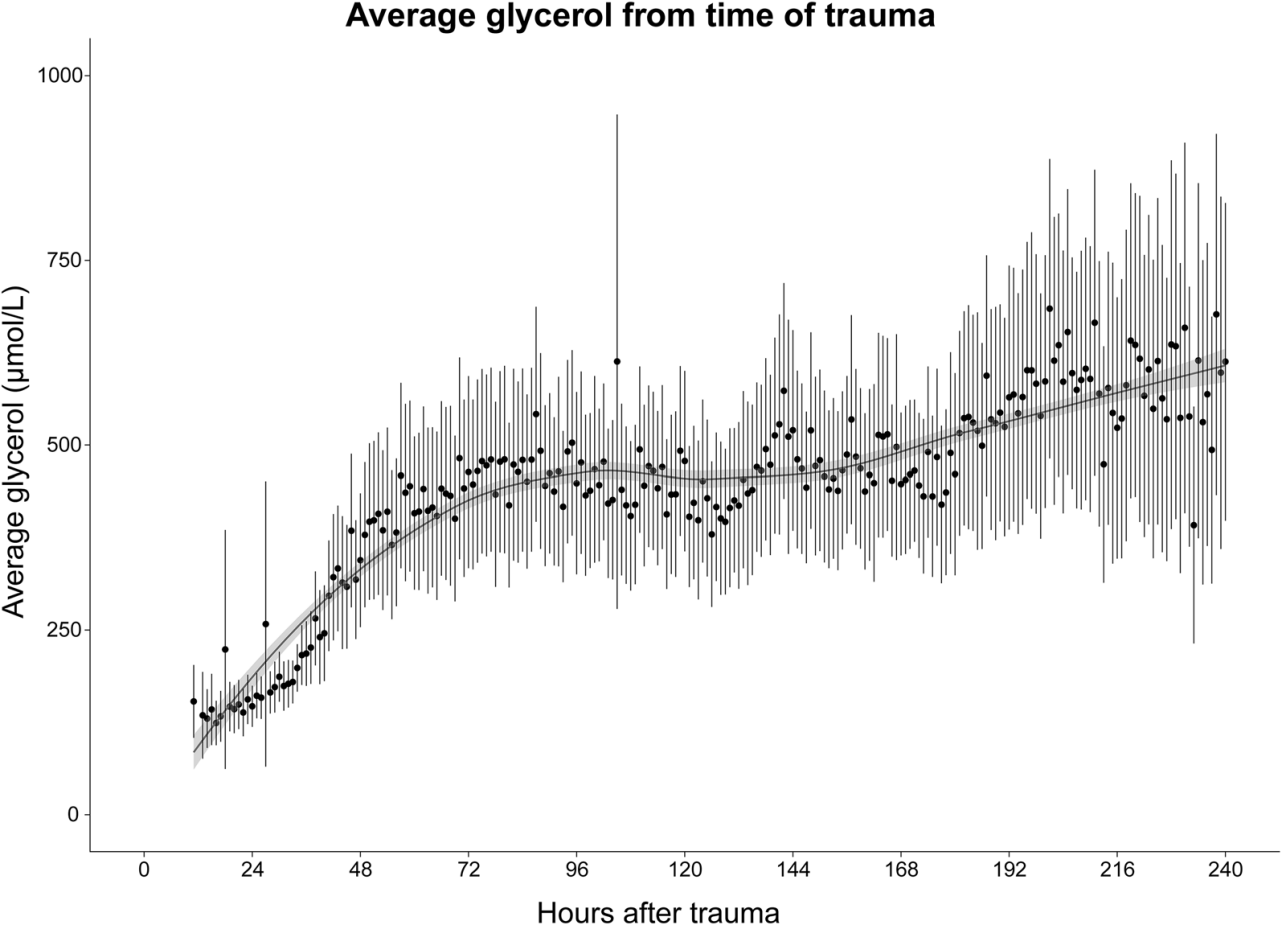
Trend in CMD-glycerol after trauma. A graph displaying the average CMD-glycerol for all TBI patients during the first 10 days after trauma. The error bars encompass the 95% confidence interval. A LOESS curve has been fitted to the data. Abbreviations: *CMD*  cerebral microdialysis, *TBI* traumatic brain injury, *LOESS* locally weighted scatterplot smoothing

As standard practice in cross-correlation analysis, the need to detrend data was considered. A general trend could be discerned from the average CMD-glycerol in neurotrauma patients (Fig. 1). It was, however, also apparent that the individual data points fluctuated around that trend. To study this more closely, we calculated a detrended CMD-glycerol time series. This was performed by first fitting a third-degree polynomial model to the average hourly CMD-glycerol shown in Fig. 1. This model was then used to determine the detrended CMD-glycerol values by calculating the difference between each neurotrauma patient’s actual CMD-glycerol values and the expected values based upon the model. The detrended CMD-glycerol time series was then used to perform the cross-correlation analyses in the same manner as the raw CMD-glycerol time series.

#### Differenced Analysis

Another standard method applied to avoid finding spurious correlations in cross-correlation analysis is differencing. This was accomplished by calculating the difference in CMD-glycerol between consecutive CMD-glycerol values for each patient. The differenced CMD-glycerol time series were then used to perform all three cross-correlation analyses in the same manner as the raw CMD-glycerol time series.

#### Subgroup Analyses

In addition to analyzing all catheters as a single group, catheters were also divided into subgroups as an exploratory hypothesis generating measure (Table 1).

**Table 1:** Catheter subgroups

	All microdialysis catheters (*N* = 187)
Graphical subgroups, *n* (%)	
Climbers	73 (39.0)
Decliners	114 (61.0)
Locational subgroups, *n* (%)	
Pericontusional/perilesional	61 (32.6)
Non-pericontusional/non-perilesional	98 (52.4)
Cerebrospinal fluid	21 (11.2)
Unknown	7 (3.7)

*N*, total number of catheters

Climbers displayed a trend of increasing cerebral microdialysis-glycerol, while decliners did not exhibit this trend. Catheters whose intracranial location could not be verified with radiological imaging were classified as unknown and not included in the analysis

**Fig. 2:**
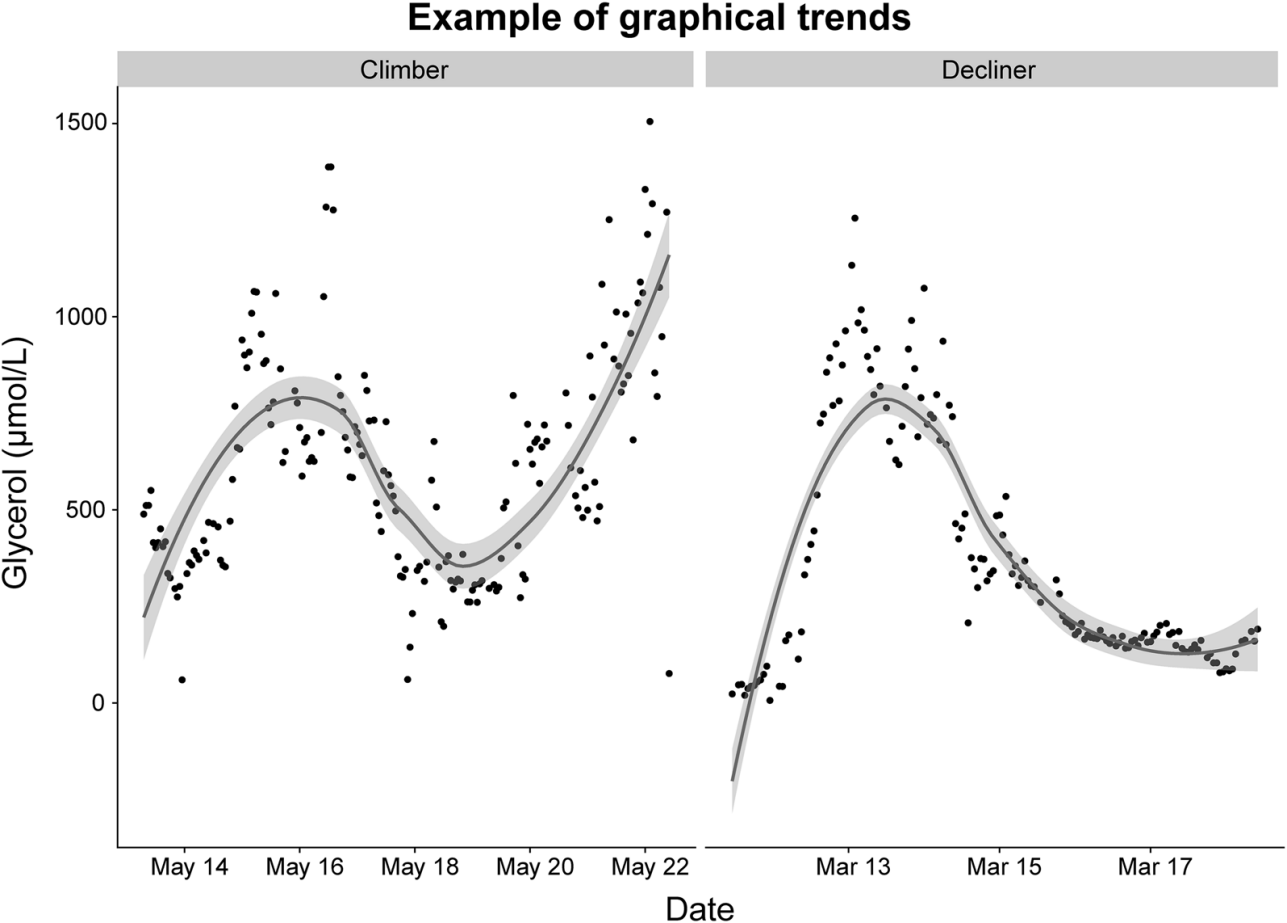
Comparison of graphical trends of CMD-glycerol. A LOESS curve has been fitted to each graph. The graph on the left displays an example of a catheter which exhibited a trend of increasing CMD-glycerol (climber). The graph on the right displays an example of a catheter which did not exhibit this trend (decliner). Abbreviations: *CMD* cerebral microdialysis, *LOESS* locally weighted scatterplot smoothing

The first set consisted of two subgroups: catheters which showed a trend of increasing CMD-glycerol (climbers) and catheters which did not (decliners) (Fig. 2). This was assessed by plotting each catheter’s CMD-glycerol over time and thereafter fitting a locally weighted scatterplot smoothing (LOESS) curve to the plot. The LOESS curve was used in conjunction with the plotted points to determine whether the patient was a climber or decliner.

The second set consisted of three subgroups: microdialysis catheters that were pericontusional/perilesional, catheters that were non-pericontusional/non-perilesional, and catheters that monitored the compounds in the cerebrospinal fluid (CSF). A catheter was defined as pericontusional or perilesional if it was located within 2 cm of a lesion (hyperdense or hypodense contusion, hematoma border, or infarction on computerized tomography [CT]-images of the brain), as per Nelson et al. [12]. Non-pericontusional/non-perilesional catheters were placed more than 2 cm from a lesion. Catheters placed in the external ventricular drain (*n* = 12) and catheters accidentally inserted into the CSF (*n* = 9) were defined as monitoring the CSF.

Predominantly, CT images were used, but in three patients no CT images of the catheter were available, so a magnetic resonance image was used instead. Seven patients had CT images where the exact location of the catheter could not be definitively determined; they were excluded from this analysis.

#### Cross-Correlation Analysis with S100B

The trend of increasing CMD-glycerol appears to be unrelated to other apparent ongoing pathology. We performed two cross-correlation analyses between serum levels of S100B and CMD-glycerol to determine whether this is a reasonable assessment; the first analysis used the raw S100B data, while the second employed S100B values which had been detrended using the function presented by Ercole et al. [13]. S100B was selected as it is routinely sampled twice daily (Elecsys^®^, Roche, Basel, Switzerland) in our NCCU to monitor patients for the occurrence of secondary cerebral damage [14]. CMD-glycerol was down-sampled by calculating the area under the curve for every 12-h interval since cross-correlation analyses require both time series to have an equal amount of time between consecutive measurements.

## Results

### Sample Population

**Fig. 3:**
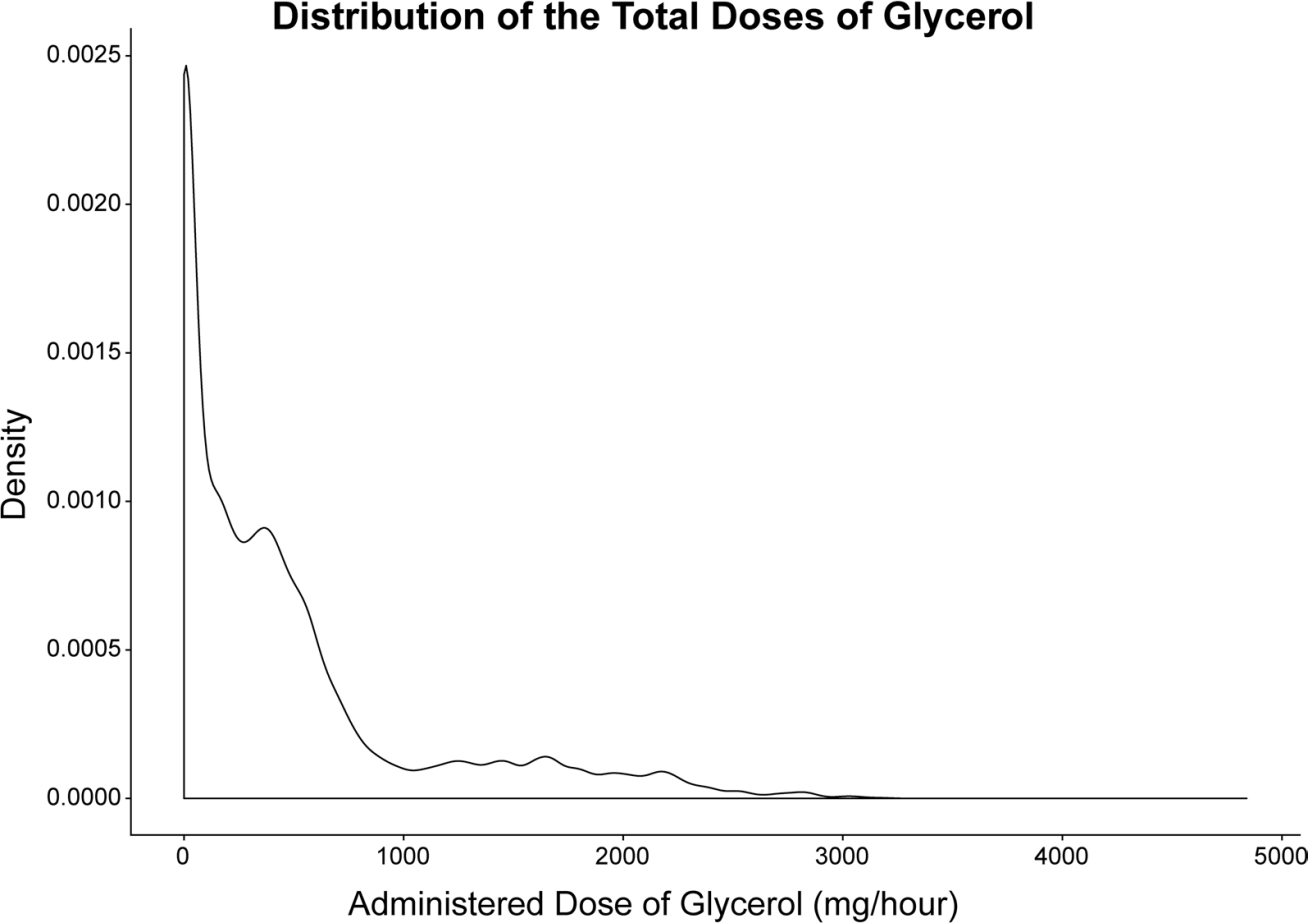
Density plot of the total ADG. The distribution of all administered doses of glycerol in mg/h. Abbreviations: *ADG* administered dose of glycerol

The sample population was 59.7% male, and patients were primarily admitted to the NCCU with a median initial GCS of 5 because of TBI (64.2%), spontaneous SAH (15.1%), as well as bacterial meningitis (13.8%). The interquartile range for the patients’ ages at catheter insertion was 33.5–62.5 years of age. The median time with CMD was 172.5 h (Table 2). The patients received an average total ADG of 470 mg/h, while the maximum dose given to any patient was 4840 mg in 1 h (Fig. 3).

**Table 2:** Description of sample population

	All patients (*N* = 159)
Median age at catheter insertion (IQR)	52 (33.5–62.5)
Sex, *n* (%)	
Male	95 (59.7)
Female	64 (40.3)
Median time with microdialysis (IQR)	172.5 (118–221.8)
Median initial GCS (IQR)	5 (3–7)
Reason for admission, *n* (%)	
Traumatic brain injury	102 (64.2)
Spontaneous subarachnoid hemorrhage	24 (15.1)
Bacterial meningitis	22 (13.8)
Intracerebral hemorrhage	5 (3.1)
Cerebral infarction	4 (2.5)
Tick-borne encephalitis	1 (0.6)
Chronic subdural hematoma	1 (0.6)

*N*, total number of patients

Age is expressed in years. Time with microdialysis is expressed in hours. Initial GCS is the first score registered in the patient data management system

*GCS* Glasgow Coma Scale, *IQR* interquartile range

### Correlation Between ADG and CMD-Glycerol

Raw CMD data did not show any correlation between the ADG and CMD-glycerol, but as raw CMD data display a high degree of autocorrelation [12], we chose to perform a detrended and differenced analysis. Detrending was only performed on the neurotrauma group as only this group presented a defined time for ictus.

#### Free ADG and Detrended CMD-Glycerol (Trauma Patients; *n* = 102)

**Fig. 4:**
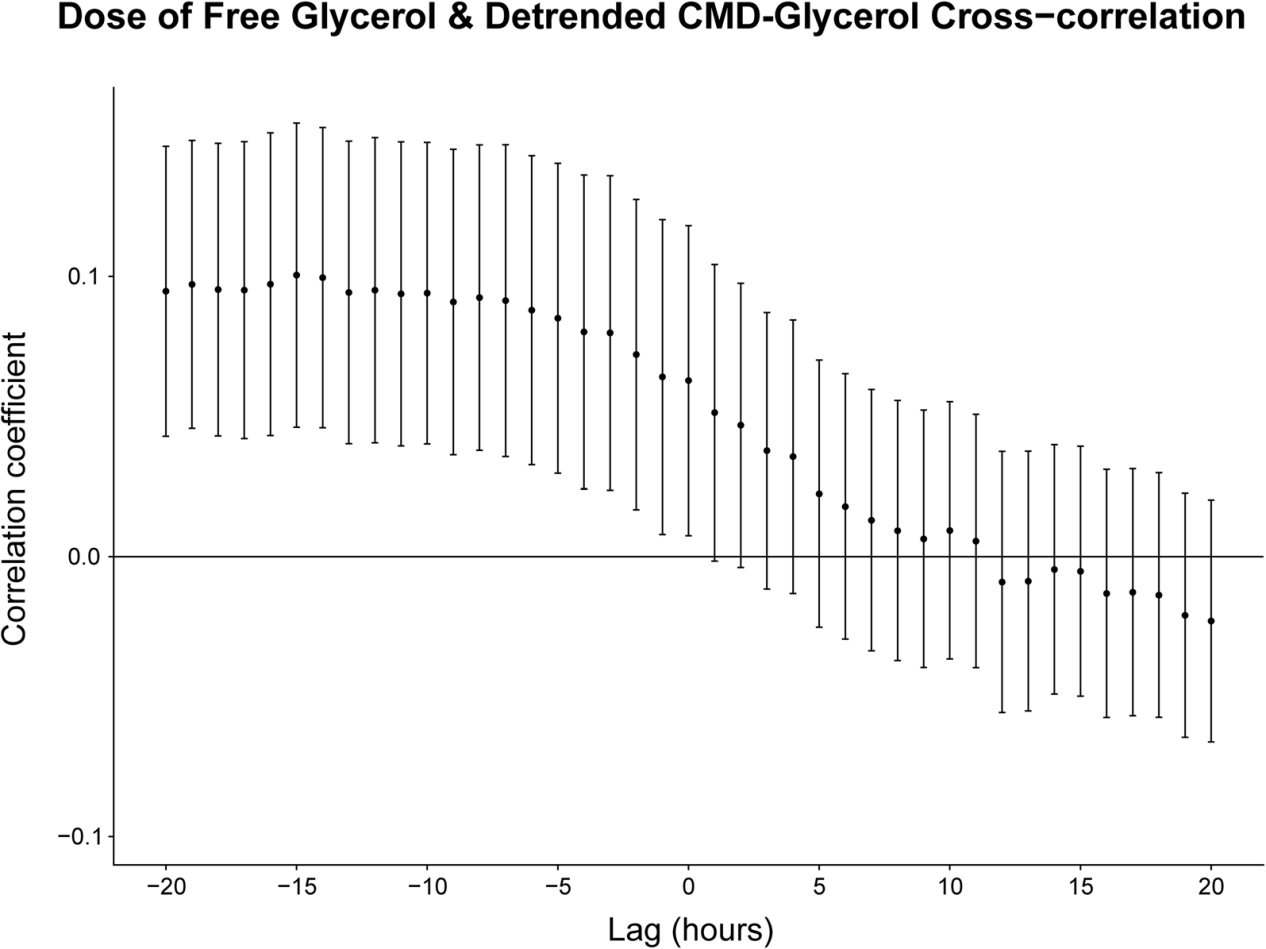
Cross-correlation analysis between the free ADG and detrended CMD-glycerol. The Pearson correlation coefficient is used. The error bars encompass the 95% confidence interval. CMD-glycerol lags the ADG for all lag values less than 0, e.g., the correlation coefficient at a lag value of − 5 corresponds to the correlation between the ADG and the CMD-glycerol values measured 5 h later. Abbreviations: *CMD* cerebral microdialysis, *ADG* administered dose of glycerol

Cross-correlation analysis showed statistically significant results for all lag values within the specified range where the detrended CMD-glycerol lags the free ADG (Lag − 20 → − 1) and where there was no lag (Lag 0), that is from the time of intravenous administration up to 20 h later (Fig. 4). This yielded a maximum correlation of 0.1 (0.05–0.15, 95% CI) at a lag of 15 h.

#### Metabolized ADG and Detrended CMD-Glycerol (Trauma Patients; *n* = 102)

**Fig. 5:**
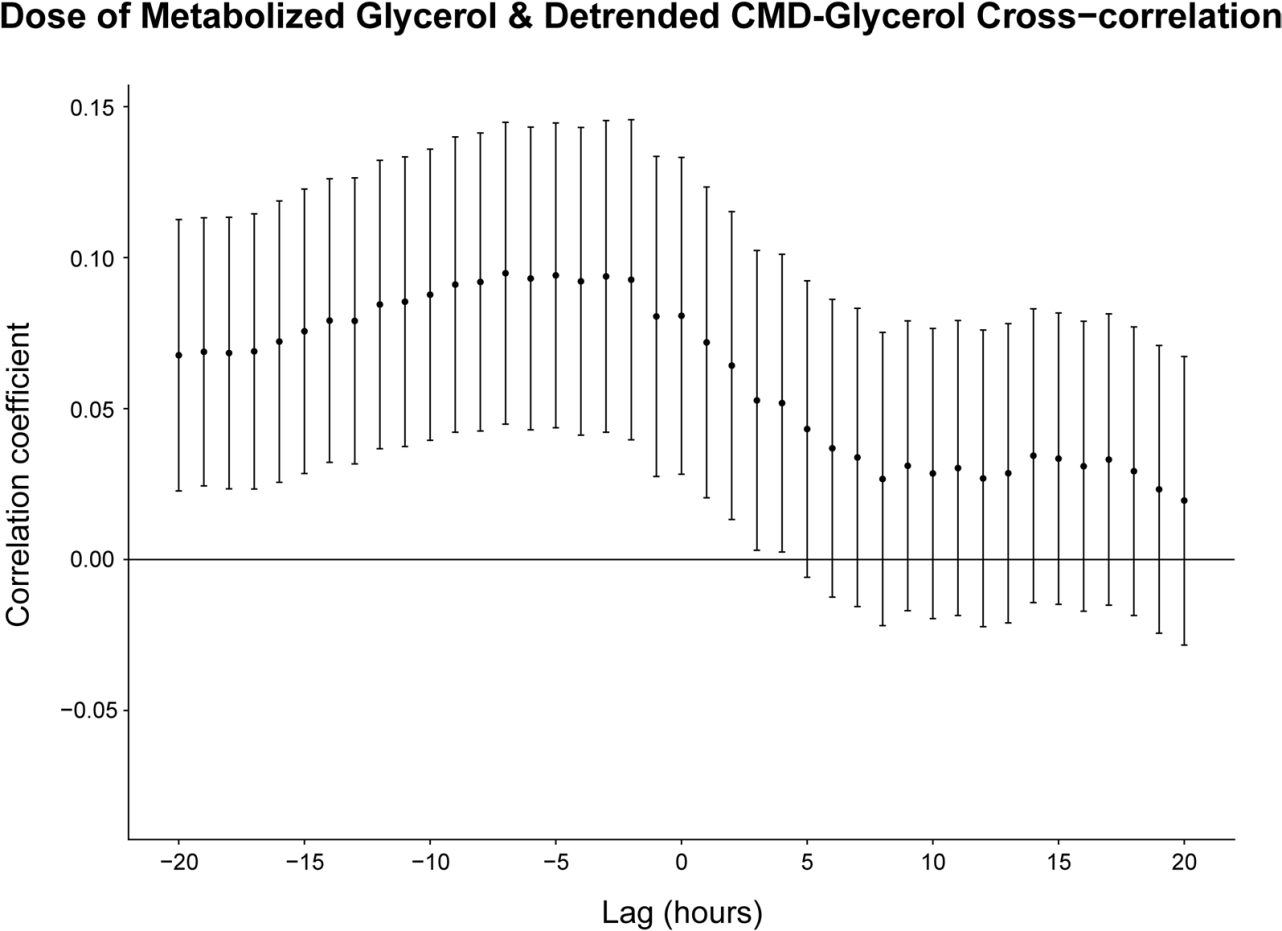
Cross-correlation analysis between the metabolized ADG and detrended CMD-glycerol. The Pearson correlation coefficient is used. The error bars encompass the 95% confidence interval. CMD-glycerol lags the ADG for all lag values less than 0, e.g., the correlation coefficient at a lag value of − 5 corresponds to the correlation between the ADG and the CMD-glycerol values measured 5 h later. Abbreviations: *CMD* cerebral microdialysis, *ADG* administered dose of glycerol

The cross-correlation analysis showed statistically significant results for all lag values within the specified range where the detrended CMD-glycerol lags the metabolized ADG (Lag − 20 → − 1), and where there is no lag (Lag 0) (Fig. 5). This yielded a maximum correlation of 0.09 (0.04–0.14, 95% CI) at a lag of 7 h.

#### Total ADG and Detrended CMD-Glycerol (Trauma Patients; *n* = 102)

**Fig. 6:**
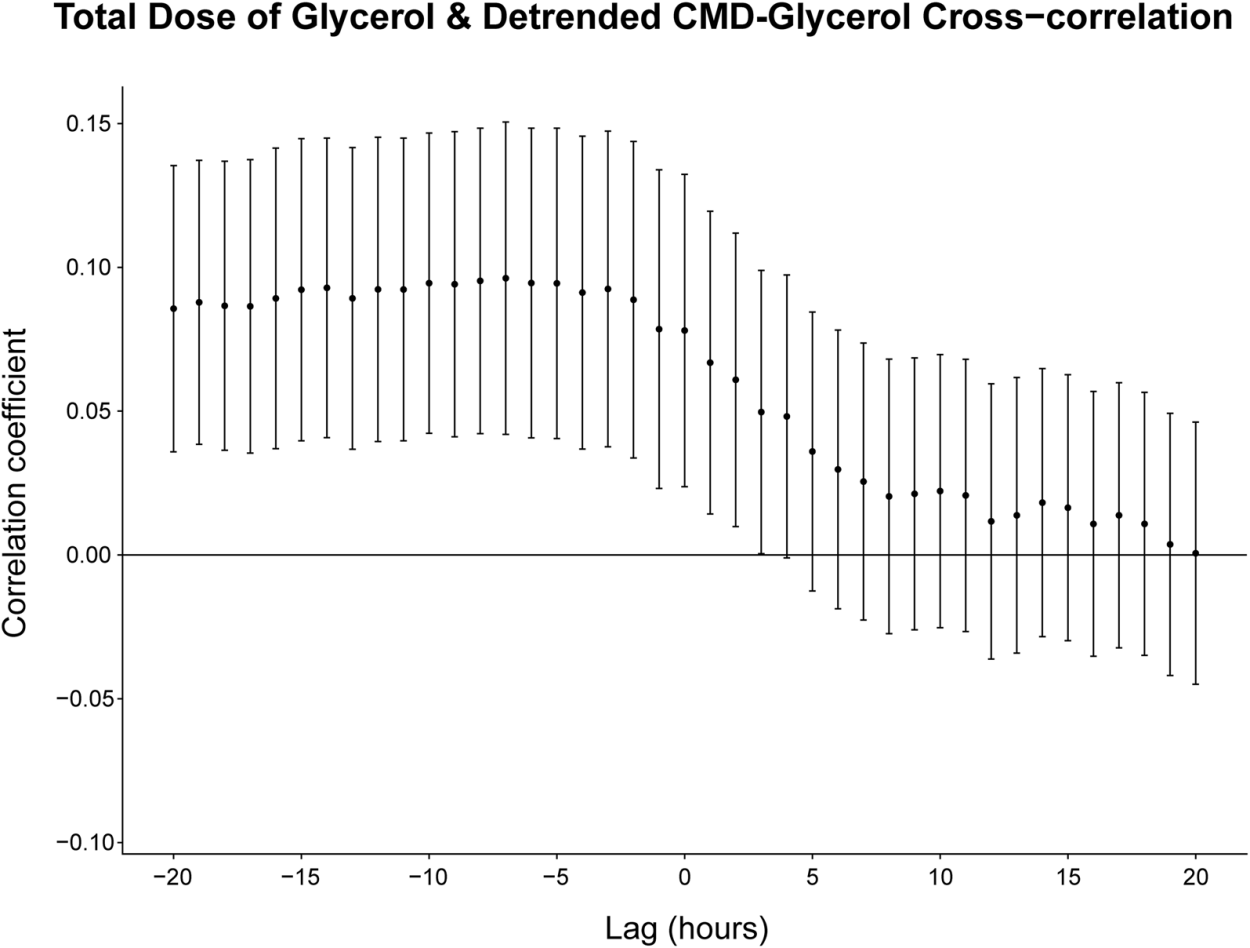
Cross-correlation analysis between the total ADG and detrended CMD-glycerol. The Pearson correlation coefficient is used. The error bars encompass the 95% confidence interval. CMD-glycerol lags the ADG for all lag values less than 0, e.g., the correlation coefficient at a lag value of − 5 corresponds to the correlation between the ADG and the CMD-glycerol values measured 5 h later. Abbreviations: *CMD* cerebral microdialysis, *ADG* administered dose of glycerol

The cross-correlation analysis showed statistically significant correlations for all lag values within the specified range where the detrended CMD-glycerol lags the total ADG (Lag − 20 → − 1) and where there is no lag (Lag 0) (Fig. 6). This yielded a maximum correlation of 0.1 (0.04–0.15, 95% CI) at a lag of 7 h.

#### ADG and Differenced CMD-Glycerol (All Patients)

Cross-correlation analyses were performed between the free, metabolized and total ADG and the differenced CMD-glycerol. None of these analyses provided any clinically significant correlations between the ADG and CMD-glycerol at any of the lag values.

### Subgroup Analyses

#### Division of Catheters

The graphical trend of increasing CMD-glycerol (climbers) was less common than other trends (decliners), but it was still seen in approximately 4 out of every 10 catheters. The microdialysis catheters were primarily located in the cerebral parenchyma with the majority of those being non-pericontusional or non-perilesional (Table 1).

#### Graphical Subgroup Analyses (All Patients)

The catheters were divided into two subgroups based upon the graphical trend of their CMD-glycerol curve. The three cross-correlations were then performed on each subgroup (climbers and decliners) for a total of six analyses. None of these analyses showed any statistically significant correlations between the ADG and CMD-glycerol at any of the lag values.

#### Locational Subgroup Analyses (All Patients)

The catheters were divided into three subgroups based upon their intracranial location (pericontusional/perilesional, non-pericontusional/non-perilesional, and CSF). The three cross-correlation analyses were then performed on each subgroup for a total of nine analyses. None of these analyses showed any statistically significant correlations between the ADG and CMD-glycerol at any of the lag values.

### Correlation Between S100B and CMD-Glycerol

No clinically significant correlations were found between S100B and the area under the CMD-glycerol curve when cross-correlation analyses were performed. Furthermore, no clinically significant correlations were found between the detrended S100B values and the area under the CMD-glycerol curve.

## Discussion

To our knowledge, this is the first study to report the effects of intravenously administered glycerol-containing drugs on the cerebral glycerol levels measured by CMD. The cross-correlation analyses using the differenced and raw CMD-glycerol time series provided no evidence of a correlation between the ADG and CMD-glycerol. Instead, statistically significant correlations were only found in analyzing the detrended CMD-glycerol time series, yet the correlations are so low that they lack clinical relevance. Furthermore, the cross-correlation analyses between CMD-glycerol and S100B suggest that pathologies that lead to an S100B increase in serum are unrelated to the trend of increasing glycerol in the ECF. The fact that S100B has been shown to correlate with lesion size in TBI and stroke [15, 16] also suggests that CMD-glycerol levels are not associated with the cerebral volume affected. Additional studies to investigate the cause of increased CMD-glycerol levels are warranted.

The lack of correlation between the ADG and CMD-glycerol stands in stark contrast to the previously mentioned case report by Gliemroth et al. [8]. However, this is not unreasonable as the patients described by Gliemroth et al. received an enema containing between 500 and 1000 mg of glycerol over a period of, at most, a couple of minutes. In contrast, the patients in this study received an average total ADG of 470 mg per hour. Even if we consider the maximum total ADG given to any patient, 4840 mg of glycerol in 1 h, this was still only equivalent to approximately 80 mg of glycerol per minute. Two case reports [8, 9] on CMD-glycerol responses following administration of glycerol-containing enemas have been published. In these, a clear relationship could also be seen in the general trend of CMD-glycerol. In aggregate, it appears reasonable that at lower doses of administered glycerol, such as in our study, the general trend obfuscates any evidence of such a correlation.

Using the detrended CMD-glycerol, the peak correlation calculated from the free ADG was observed with a lag of 15 h while the peak correlation calculated from the metabolized ADG was observed with a lag of 7 h. This essentially implies that, for the free glycerol, 15 h elapsed between its administration and reaching its maximal concentration in the cerebral ECF, while only 7 h elapsed between the administration glycerol-containing compounds and the glycerol released from their metabolism reaching its maximal concentration in the cerebral ECF. This does not seem rational considering that the free glycerol does not need to be metabolized like the glycerol-containing compounds. Additionally, the half-life of intravenously administered glycerol has been estimated to be 1.03–3.68 h in CSF [17] and Gliemroth et al. saw a glycerol peak after only 3–5 h [8]. The detrended CMD-glycerol time series also still displayed a high degree of autocorrelation despite the detrending. Since the ADG time series are also autocorrelated, this results in a high risk that the observed weak correlations merely are the result of two strongly autocorrelated, yet independent, time series rather than a reflection of an actual causal relationship, as described by Dean et al. [18]. In summary, the above leads us to believe that the, weak albeit identified, correlations in our study are spurious and conclude that no significant relationship was seen within the expected time frame.

The location of the microdialysis catheter plays an integral role in the interpretation of concentrations measured [1, 19, 20]. Accordingly, we divided the catheters into groups based upon their location. Damage to the BBB can be expected to exist more in pericontusional [21, 22] and perilesional [23–26] tissue. Since glycerol does not pass freely through the BBB [7], damage could be inferred to increase the permeability, thereby allowing a larger fraction of the ADG to pass through to the cerebral ECF. Nonetheless, the cross-correlation analyses revealed no indication of this which demonstrates that location does not play a significant role in the effect of the ADG on CMD-glycerol at the therapeutic doses received in the NCCU.

Initially, we could not eliminate the risk that a larger proportion of the ADG was reaching the cerebral ECF in a subset of patients due to some unknown factor pertaining to that group. By performing similar analyses on the graphical subgroups, we determined that this did not appear to be the case. Neither subgroup showed any statistically significant correlations in any of the cross-correlation analyses.

### Strengths and Limitations

Most of the strengths and limitations in the study can be derived from the use of retrospective data. The primary advantage was the relatively large size of the dataset. It provided accurate doses for all drugs patients received intravenously, and we could therefore be reasonably assured that we accounted for all intravenous sources of glycerol. The dataset also had a high temporal resolution. The infusion rate for each drug was recorded, at the very least, once every hour in addition to every time the infusion rate was changed. Furthermore, these values were recorded automatically in the PDMS, which reduced the risk of human error.

The most prevalent limitation was missing data, primarily concerning the microdialysis time series. Employees at the NCCU had to manually insert vials into the microdialysis analyzer for the data to be sent to the PDMS. Occasionally, this did not happen, which resulted in an hour without a value for the CMD-glycerol. Besides missing data, the dataset also contained noticeably incorrect values often identifiable as data insert errors and mismatch. Fortunately, these were exceedingly rare. These errors were identified and recorded before being removed or replaced (i.e., switched to the correct value) as was deemed prudent. Additionally, we could not control for other factors which could have affected CMD-glycerol levels, such as sources of glycerol administered orally, e.g., enteral nutrition, or rectally. However, we do administer glycerol-containing enemas in our unit, but the contribution from that and other orally administered drugs is expected to be minimal.

Another weaknesses were the assumptions required for calculating the metabolized dose of glycerol: (1) All the glycerol-containing compounds in the drug were metabolized to glycerol. (2) The maximum amount of glycerol possible was released from each compound. (3) All released glycerol ended up in the systemic circulation, and (4) All compounds were metabolized at approximately the same rate. The purpose behind these assumptions was to calculate the maximum amount of glycerol that could possibly have been released by metabolism of the glycerol-containing compounds in the drugs. It was, however, very likely that patients deviated from these assumptions.

### Clinical Significance

According to the 2014 consensus statement [4], CMD-glycerol has been known to be affected by the administration of glycerol-containing substances. Despite this, our results indicate that the doses supplied through routine infusions and injections at the NCCU do not appear to have a significant effect on CMD-glycerol. Accordingly, we propose that clinicians do not need to take routine infusions into account when interpreting the CMD-glycerol level. Care should, however, be taken when administering drugs in which glycerol is the primary active substance as the higher doses present in these drugs may still be enough to affect the CMD-glycerol level.

### Future Studies

It is possible that the actual insertion of the catheter is causing damage to surrounding cells initiating cell death and cell membrane decomposition, with a subsequent increase in local interstitial glycerol concentration. In a previous review, CMD has been shown to cause microscopic bleeding and edema in the adjacent tissue in addition to macrophage infiltration and astrocyte hypertrophy in animal models, but only minimal findings or no lesions at all in postmortem human studies [27]. Future studies should focus on alternative causes of cell membrane decomposition that results in the elevation of the cerebral glycerol concentration.

Another potential reason for increased glycerol could be that it is absorbed from the catheter membrane. However, following discussion with the manufacturer (µDialysis AB, Stockholm, Sweden), this appears unlikely in this case as the membrane is composed of polyamide to avoid such a problem occurring.

## Conclusion

Administered glycerol in the NCCU does not seem to be correlated with the CMD-glycerol level. While detrending generated some positive correlations, we do not believe this is clinically relevant; the doses routinely administered in the NCCU are too low to create a clinically relevant correlation between the ADG and CMD-glycerol.
